# An In Silico Study of the Antioxidant Ability for Two Caffeine Analogs Using Molecular Docking and Quantum Chemical Methods

**DOI:** 10.3390/molecules23112801

**Published:** 2018-10-29

**Authors:** Josivan da Silva Costa, Ryan da Silva Ramos, Karina da Silva Lopes Costa, Davi do Socorro Barros Brasil, Carlos Henrique Tomich de Paula da Silva, Elenilze Figueiredo Batista Ferreira, Rosivaldo dos Santos Borges, Joaquín María Campos, Williams Jorge da Cruz Macêdo, Cleydson Breno Rodrigues dos Santos

**Affiliations:** 1 Postgraduate Program in Biotechnology and Biodiversity-Network BIONORTE, Federal University of Pará, Rua Augusto Corrêa, 01, Belém, Pará 66075110, Brazil; josivan.chemistry@gmail.com (J.d.S.C.); lqfmed@gmail.com (R.d.S.B.); williams.macedo@ufra.edu.br (W.J.d.C.M.); 2 Laboratory of Modeling and Computational Chemistry, Department of Biological Sciences, Federal University of Amapá, Rod. Juscelino Kubitschek, Km 02, s/n, Macapá, Amapá 68902-280, Brazil; ryanquimico@hotmail.com (R.d.S.R.); karinalopesfarm@gmail.com (K.d.S.L.C.); elenilze@yahoo.com.br (E.F.B.F.); 3 Laboratory of Molecular Modeling and Simulation System, Federal Rural University of Amazônia, Rua João Pessoa, 121, Capanema, Pará 68700-030, Brazil; 4 Institute of Technology, Federal University of Pará, Av. Augusto Corrêa, 01, Belém, Pará 66075-900, Brazil; davibb@ufpa.br; 5 Computational Laboratory of Pharmaceutical Chemistry, Faculty of Pharmaceutical Sciences of Ribeirão Preto, São Paulo 14040-903, Brazil; tomich@fcfrp.usp.br; 6 Department of Pharmaceutical and Organic Chemistry, University of Granada, Campus of Cartuja, 18071 Granada, Spain; jmcampos@ugr.es

**Keywords:** antioxidant potential, molecular descriptors, molecular docking, binding free energy, free radicals, oxidative stress

## Abstract

The antioxidant activity of molecules constitutes an important factor for the regulation of redox homeostasis and reduction of the oxidative stress. Cells affected by oxidative stress can undergo genetic alteration, causing structural changes and promoting the onset of chronic diseases, such as cancer. We have performed an in silico study to evaluate the antioxidant potential of two molecules of the zinc database: ZINC08706191 (Z91) and ZINC08992920 (Z20). Molecular docking, quantum chemical calculations (HF/6-31G**) and Pearson’s correlation have been performed. Molecular docking results of Z91 and Z20 showed both the lower binding affinity (BA) and inhibition constant (Ki) values for the receptor-ligand interactions in the three tested enzymes (cytochrome P450—CP450, myeloperoxidase—MP and NADPH oxidase—NO) than the control molecules (5-fluorouracil—FLU, melatonin—MEL and dextromethorphan—DEX, for each receptor respectively). Molecular descriptors were correlated with Ki and strong correlations were observed for the CP450, MP and NO receptors. These and other results attest the significant antioxidant ability of Z91 and Z20, that may be indicated for further analyses in relation to the control of oxidative stress and as possible antioxidant agents to be used in the pharmaceutical industry.

## 1. Introduction

Oxidants play a key role in maintaining the redox homeostasis of cells. However, in large quantities an imbalance can be triggered. Reactive Oxygen Species (ROS) resulting from aerobic respiration are examples of extremely unstable oxidants that can collide with other species (molecules or biomacromolecules), causing their transformation (oxidative damages) and the increased oxidative damage causes cell stress, known as oxidative stress [1,2].

Several chronic diseases such as diabetes, neurodegenerative and cardiovascular diseases, and cancer can be caused by increased oxidative stress [3]. This occurs from the activation of various transcription factors, which can express hundreds of different genes, such as growth factor promoters and inflammatory cytokines, which lead to the activation of inflammatory pathways transforming a normal cell into a cancerous one [4].

Thus, maintenance of redox homeostasis and reduction of oxidative stress depend on the efficiency of antioxidant present in the cell, since the first and second defense barriers (antioxidant enzymes and proteolytic and lipolytic enzymes, respectively) have already been overcome [5]. Some enzymes, such as cytochrome P450 (CP450), lipoxygenase (LO), myeloperoxidase (MP), NADPH oxidase (NO) and xanthine oxidase (XO) that are known to generate ROS during the metabolism of arachidonic acid and their inhibitions break the ROS production cycle with the consequent reduction of the oxidative stress and maintenance of redox homeostasis [6]. The increase of the oxidative stress mediated by ROS may lead to the appearance of several diseases, including cancer. Therefore, the search of agents that maintains the balance of redox homeostasis (antioxidants) has an important role in the discovery of molecules that prevent and halt the growth of cancer cells via reduction of the oxidative stress [3,4,5,6].

The literature shows that molecular docking has been an important tool for studies of receptor-ligand interaction in the inhibition of enzymes related to antioxidant activity. This technique has clarified doubts and pointed out clarifications about the possible region of the receptor where the activity occurs, what amino acid residues are involved in the interactions and what atoms are directly interacting with the ligand [7].

Molecular docking has aided in the elucidation of the antioxidant mechanism of compounds submitted to biological tests such as new chromeno-carbamodithioates derivatives. These compounds have been evaluated for their antioxidant activity in the cyclooxygenase-2 enzyme, using the GOLD program to assess the full range of ligand flexibility and the rotational flexibility of selected receptor hydrogens, and the Autodock 4.0 program to check out the binding free energy and inhibition constant (Ki) concerning the interaction of ligands with the receptor [8]. Molecular docking study at the PPARα/γ receptor has been used to evaluate the agonist and the antioxidant activity of a novel structural class of coumarin-chalcone fibrates using AutoDock 4.2.6 program [9].

As well as molecular docking analysis, significant importance can be attributed to obtaining molecular descriptors and information obtained from chemical-quantum calculations. These data assist in the elucidation of various physical and chemical properties resulting from the different classes of existing compounds [10]. A study on the antioxidant activity of 4-hydroxyphenyl substituted thiopyrimidines has been performed using the Gaussian 9 program to obtain the energies of molecular orbitals (HOMO e LUMO) and evaluation of the electron donor and acceptor character of the chemical species under analysis [11].

In similar studies, the antioxidant activity was evaluated for 1,3,4-thiadiazole derivatives [12] and for major chemical constituents present in the leaves of the *Curatella americana* Linn [13]. In both studies, molecular descriptors such as the dipole moment, polarizability, chemical hardness/softness, electronegativity and molecular orbital energies have been calculated, analyzed and the results used to evaluate the reactivity and stability of the species studied in relation to antioxidant activity.

In this manuscript, two caffeine analogs of the zinc database ZINC08706191 (Z91) and ZINC08992920 (Z20), proposed by Costa et al. (2018) [14], have been tested in silico to evaluate the antioxidant potential via molecular docking with five enzymes (CP450, LO, MP, NO and XO). They have been compared with known molecules that were used as positive controls, such as: 5-fluorouracil (FLU), zileuton (ZIL), melatonin (MEL), dextromethorphan (DEX) and febuxostat (FEB), for each receptor, respectively. Quantum chemical calculations (HF/6-31G**) and Pearson’s correlation were performed for compounds studied here.

## 2. Results and Discussion

### 2.1. Evaluation of Molecular Docking

**Figure 1:**
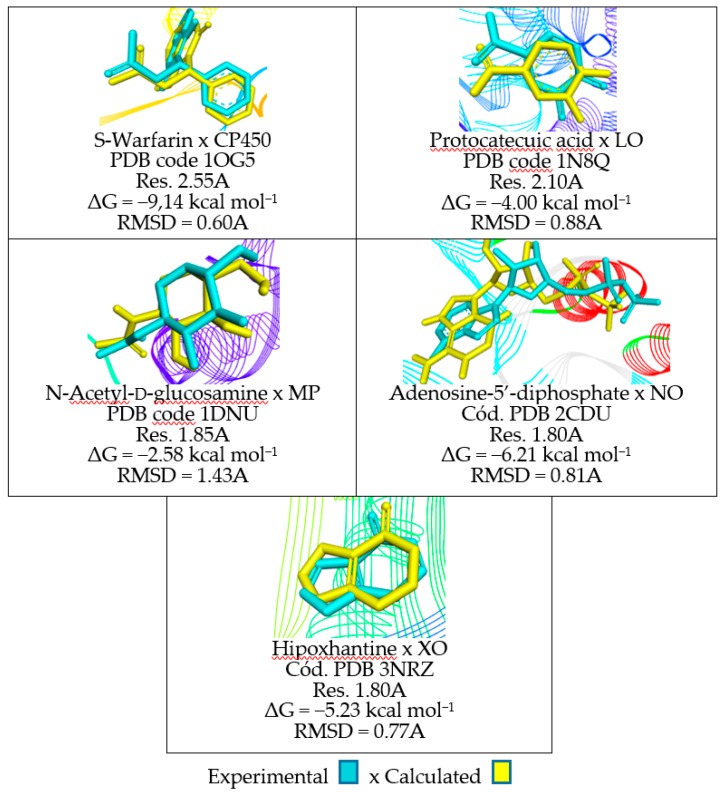
Data obtained in the validation of the molecular docking protocols for the receptors cytochrome P450 (CP450), lypoxygenase (LO), myeloperoxidase (MP), NADPH oxidase (NO) and xanthine oxidase (XO).

Data about validation protocols for molecular docking can be seen in Figure 1. According to literature, the RMSD values expressing the relationship between the calculated X crystallographic data of the complexed ligand must be less than 2.0 Å [14,15,16]. The similarity in the overlapping of crystallographic poses (orientation + conformation, cyan) and calculated (yellow) was obtained via molecular docking and graphically displays a low RMSD value, what characterizes good results according to literature (see Figure 1). These results attest that the protocols used can be applied in the molecular docking analyzes between the receptors of the antioxidant activity and ligands.

**Figure 2:**
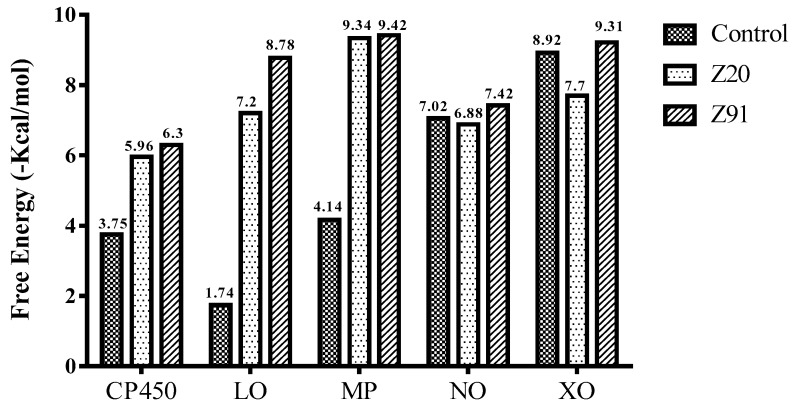
Binding free energy values resulting from the molecular docking between the molecules and receptors evaluated. Control molecules for CP450, LO, MP, NO and XO were 5-fluorouracil (FLU), zileuton (ZIL), melatonin (MEL), dextromethorphan (DEX) and febuxostat (FEB), respectively.

The binding free energies (ΔG) for the molecules evaluated at each receptor are shown in Figure 2, and these values were used to classify the best poses obtained in the molecular docking analyses. Only the smallest ΔG values for the best poses are shown. The larger the peaks, the lower the ΔG and consequently the more significant the interaction between the receptor and the ligands for the antioxidant ability.

**Figure 3:**
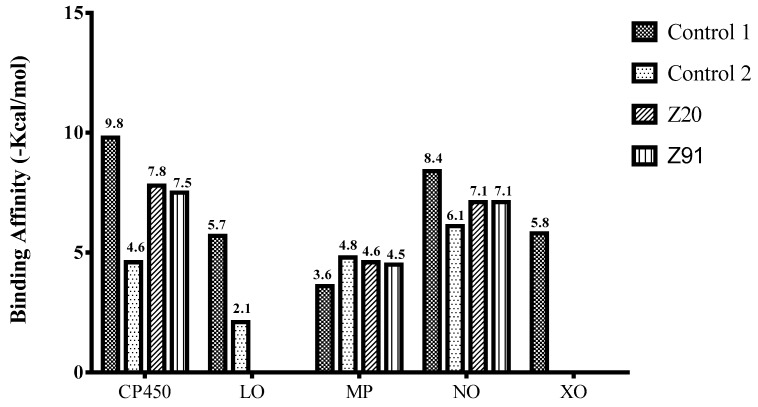
Binding affinity provided by AutoDock/Vina software of the tested molecules (Z20 and Z91). Control 1/Control 2 molecules for CP450, LO, MP, NO and XO were FLU/S-warfarin, ZIL/protocatechuic acid, MEL/*N*-acetyl-D-glucosamine, DEX/adenosine-5’-diphosphate and FEB/hypoxanthine, respectively.

Figure 3 shows binding affinity data values for the selected molecules from the ΔG values. The tested molecules (Z91 and Z20) showed very similar binding affinity levels at the CP450, MP and NO receptors. The binding affinity values at these three receptors for the molecules tested are higher than control 1 and lower than control 2. The molecules tested had values of non-significant binding affinities (positive—not shown) in the LO and XO receptors, and these were excluded to subsequent analyzes.

**Figure 4:**
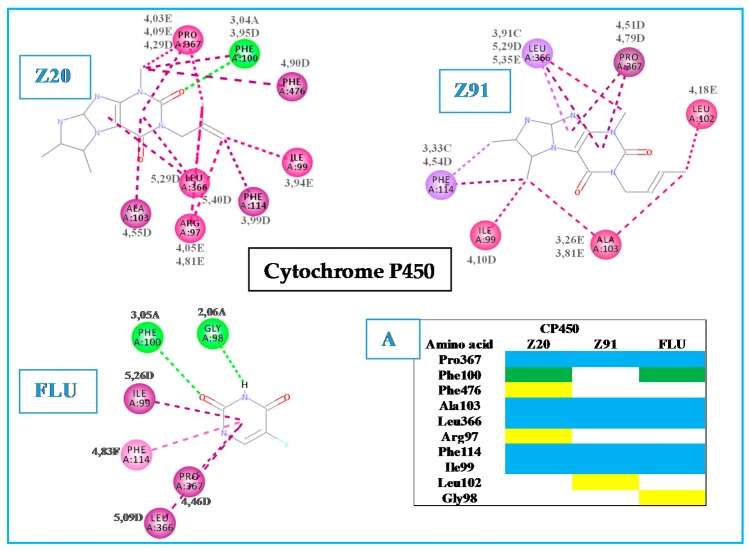
Interactions of the tested molecules (Z20 and Z91) and control (FLU) with the CP450 receptor. In A, interactions common to the three ligands (blue), two ligands (green) and interactions presented by a single ligand (yellow) are presented. 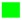 Hydrogen bond (A); 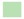 carbon-hydrogen bond (B); 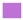 pi-sigma (C); 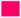 pi-alkyl (D); 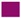 alkyl (E).

Figure 4 shows the interactions data of the tested (Z20 and Z91) and control (FLU) molecules with the CP450 receptor. Five amino acid residues (PRO367, ALA 103, LEU366, PHE 114 and ILE99) were common to Z20, Z91 and FLU (indicated by the blue in diagram A). This shows the degree of correspondence between the control and the molecules tested inferring that these may have potential antioxidant ability. The Z20 and Z91 molecules with lower number of interactions (seven and six, respectively) showed a BA (−7.8 kcal mol^−1^ and −7.5 kcal mol^−1^, respectively) higher than control 1 (FLU) with BA = −9.8 kcal.mol^−1^ (eight interactions). It is possible to verify the tendency of the BA value to decrease on increasing the number of interactions.

Additionally, study with CP450 structure complexed with *S*-warfarin shows that the active site of interaction is coated by ARG97, GLY98, ILE99, PHE100, LEU102, ALA103, VAL113, PHE114, ASN217, THR364, SER365, LEU366, PRO367 and PHE476 residues. Specific interactions occur between some of these residues and the phenyl group of *S*-warfarin (control 2), which bundles against the side chains of PHE476 (pi-pi interaction), PHE100 and ALA103 (hydrogen bond) and interact with PRO367 too [17]. Figure 4 displays a high similarity of interactions with the CP450 receptor between the tested and control molecules, and findings of the literature [17]. These results for the tested molecules together to good BA values (differences of −2.0 kcal mol^−1^ in Z20 and −2.3 kcal mol^−1^ in Z91, in relation to the FLU control) indicate a good antioxidant ability to these molecules.

**Figure 5:**
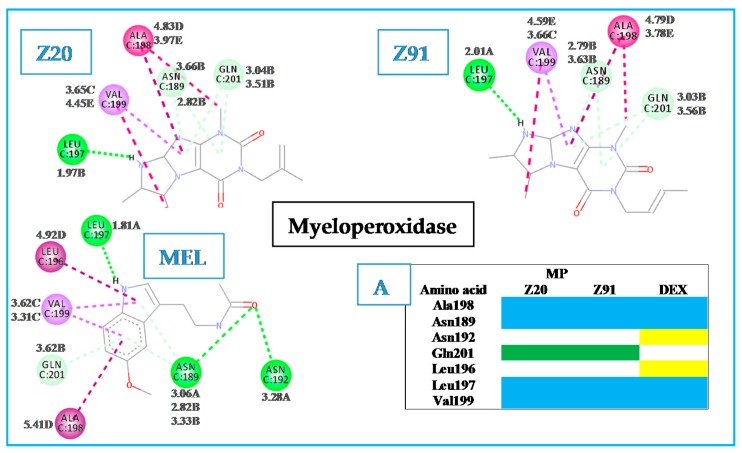
Interactions of the tested molecules (Z20 and Z91) and control (MEL) with the MP receptor. In A, interactions common to the three ligands (blue) and two ligands (green). 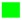 Hydrogen bond (A); 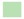 carbon-hydrogen bond (B); 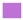 pi-sigma (C); 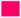 pi-alkyl (D); 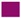 alkyl (E).

In the MP receptor (Figure 5), a maximum of six interactions were observed. In the PDB file (1DNU) [18] the active site of attachment has its location pointed out to the ASN192, GLN201 and VAL199 amino acid residues, which interact with the ligand *N*-acetyl-D-glucosamine. This ligand is complexed with the MP receptor available in the PDB (control 2, Figure 3). The three previously mentioned residues interact with the control molecule, MEL and two of them (GLN201 and VAL199) interact with Z20 and Z91, which indicates a reasonable antioxidant ability to the molecules tested. Other indications of good antioxidant ability are the similarities with the control molecule: Z20 and Z91 interact with four amino acid residues (blue in diagram A of Figure 5) common to the control and BA values lower than that of the control (−4.6 kcal mol^−1^ for Z20, −4.5 kcal mol^−1^ for Z91 and −3.6 kcal mol^−1^ for the MEL control).

**Figure 6:**
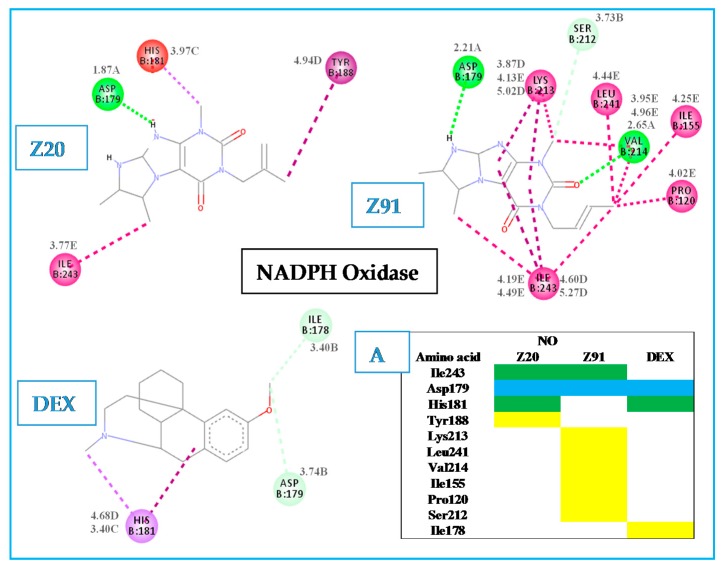
Interactions of the tested molecules (Z20 and Z91) and control (DEX) with the NO receptor. In A, interactions common to the three ligands (blue), two ligands (green) and interactions presented by a single ligand (yellow) are presented. 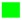 Hydrogen bond (A); 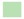 carbon-hydrogen bond (B); 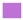 pi-sigma (C); 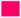 pi-alkyl (D); 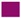 akyl (E).

In NO (see Figure 6) the ASP179 residue only showed interaction with all molecules (blue in diagram A). For this receptor an increase in the number of interactions provided an increase in the BA value, when compared to Z91 (eight interactions and BA = −7.1 kcal.mol^−1^), and with DEX control (three interactions and BA = −8.4 kcal.mol^−1^). These BA values are relatively close (difference of −1,3 kcal mol^−1^ for Z20 and Z91) to the DEX control, which indicates a good antioxidant ability of the tested molecules in the NO receptor.

Another evidence of this antioxidant ability is the similarity of the interactions and of the active site with results obtained from the literature [19]. In such study, the active site is surrounded by ILE160, ILE243, ASP179, LYS213, VAL214 and TYR188 residues, which interact with the ligand adenosine-5’-diphosphate (Control 2 in the NO receptor—Figure 3). From the list of residues, four interact with Z91 (ILE243, ASP179, LYS213 and VAL214), three with Z20 (ILE243, ASP179 and TYR188) and one with the DEX control (ASP179).

The antioxidant abilities of the control molecules may be related to the amino acid residues with which molecules interact. For this reason, the observation of the common residues and BA values close to the tested molecules, controls and literature infer that the tested molecules can have a good antioxidant ability. This relationship is very evident for both Z20 and Z91 that have high similarity in the presented characteristics (among themselves, compared to controls and BA values). This can be explained by the high structural similarity of Z20 and Z91, with evident difference only in the four-carbon radical connected to nitrogen, showing that the alkenyl group (Z91) and the vinyl group are attached to the second carbon of radical (Z20, see Table 1).

### 2.2. Molecular Descriptors and Pearson Correlations

Table 2 shows the data of the molecular descriptors and correlations with the Ki values for the three receptors analyzed (CP450, MP and NO). Ki (inhibition constant) is the concentration of the inhibitor needed to reduce the activity of the receptor by half. It reflects the binding affinity of the inhibitor with a specific receptor. The lower Ki value, the smaller the amount of inhibitor needed to reduce the reaction rate (inhibit the reaction) and the better the binding affinity [20]. The tested molecules Z20 and Z91 presented lower Ki values than the control molecules, which proves good antioxidant abilities for the tested molecules.

The molecular descriptors presented reasonable results of correlations with the Ki values (between 0.65 and 0.99) for all descriptors in the CP450 receptor. Already the NO receptor presented four significant correlations (between 0.48 and 0.74), while the MP receptor had two significant correlations (both 0.60). Relatively low correlation values may be considered non-significant according to the literature [21,22]. This shows that some of these descriptors have significant relation (values in bold) with the Ki values, (Table 2), reaffirming again the notable antioxidant ability.

Total surface area (TSA) of a molecule is related to its solubility and the molecular interaction that it can performed due to the superficial contact with other molecules [23]. Similar analysis can be done for the molar volume (MV), because the higher the TSA and MV, the higher the level of interaction. From TSA and MV the intermolecular forces operate, giving rise to the energy of molecular interaction [24]. The Ki values showed significant and negative correlations with TSA and MV only in CP450 and NO, indicating that the increase in these descriptors values is important for the Ki to decrease (increased antioxidant ability) in the mentioned receptors.

Molecules formed by different atoms have a polar covalent bond that can be quantified by a dipole moment. This is described with a positive partial charge close to a negative partial charge of the same absolute value, and the measure of the magnitude of these charges is given in Debyes (D). In the case of polyatomic molecules, a total dipole moment (TDM) is observed. The higher the TDM, the greater the polarity of the molecule and consequently greater a tendency to dissolve and interact in polar environment [25,26]. The electronegativity (χ), is directly related to TDM, and is described as the capacity that an atom has to attract to itself electrons of a bond that it makes with another atom [27]. It can be used to estimate the tendency of a molecule to attract electrons from another molecule with which it performs interaction [28].

The Ki values showed significative negative (inversely proportional) correlations with χ only the CP450 and MP receptors. The χ values increase with the Ki values to decrease. An increase in the χ promotes an increase in the polarity with consequent increase in attractive forces (between receptor and other amino acid residues) in certain regions providing better receptor-ligand interaction—Ki (good binding affinity for antioxidant ability).

The molecular hardness (η) and softness (1/η) also are important parameters that describe the reactivity of a molecule. The softness is related to basicity and to electron donation with high polarizability and low electronegativity, besides favoring the molecular flexibility with consequent chemical reactivity [29,30,31]. The hardness is already characterized by high ionization potential and high electronegativity, favoring the molecular stiffness with consequent chemical stability [32,33].

The descriptors η and 1/η presented significant values of Ki knockouts only at CP450 and NO receptors. In these receptors the 1/η values presented negative correlations with Ki, while the correlations between η and Ki were positive. Decrease of Ki with increase of the softness and decrease of the hardness, indicates the reactivity of the tested molecules and their contribution to the antioxidant ability (significant correlations with Ki).

Chemical potential (μ) of a species is expressed as a function of thermodynamic quantities. And it can be described as a form of energy absorbed or released from a chemical reaction or change of state. It is related to free energy, binding affinity and inhibition constant, being influenced by the number of atoms or molecules that are added or subtracted from the system [34,35,36]. Negative values of correlation between μ and Ki were significant only for CP450 and MP. These negative values show that the higher the values of μ, the lower the Ki values (higher values of μ favor the antioxidant ability). The tested molecules presented values of μ close to those of the control molecules, favoring the antioxidant ability in the two mentioned receptors.

### 2.3. Molecular Orbitals and Characteristic of Antioxidant Ability

Table 3 shows data and representations of the molecular orbitals, GAP values (energy variation between frontier orbitals) between all the energy states GAP_1_, GAP_2_, GAP_3_ and GAP_4_. The molecular orbitals LUMO and LUMO +1 have a direct relation with the electron affinity of a molecule, which is related to susceptibility to nucleophilic attacks [37]. The values of LUMO and LUMO+1 for Z91 and Z20 were close to those of the control molecules. This show that Z91 and Z20 are less susceptible to attack by nucleophiles. The values of LUMO and LUMO+1 were positively correlated with the Ki values. Only CP450 receptors (0.91 for LUMO and 0.88 for LUMO+1) and MP (0.54 for LUMO and 0.60 for LUMO+1) showed significant correlation values, revealing the tendency of the Ki values to decrease with the reduction of LUMO and LUMO+1 values for these receptors.

Significant correlation values with Ki were obtained for molecular orbital data (see Table 4). The HOMO orbital showed a significant correlation with Ki only in the MP receptor (0.47) and the HOMO-1 orbital only in MP (0.81) and NO (0.92). The energies of the HOMO and HOMO-1 molecular orbitals are strongly related to the ionization potential of a molecule and are indicative of the nucleophilic character of the species [38,39,40]. The results for HOMO (MP) and HOMO-1 (MP and NO) presented by Z91 and Z20 were close to those of the control molecules. These data show that as well as the control molecules, Z91 and Z20 have a higher electron donor character and greater ability to perform nucleophilic attacks. The negative correlations between HOMO and HOMO-1 orbitals and Ki values attest to the inversely proportional relation of these variables, with a decrease in Ki values from the increase in HOMO and HOMO-1 values. These results attest to the strong relationship of the molecular orbitals data with the Ki values (in this study representing the characteristic of antioxidant ability).

The energy variation between the HOMO and LUMO orbitals is called the GAP. GAP is an important indicator of the chemical reactivity of a molecule. The lower GAP values the greater chemical reactivity of the molecule and higher values the greater stability [41]. According to studies carried out by Fujishima et al. molecules with antioxidant potential are less stable and therefore, more reactive, i.e., a large gap implies a good thermodynamic stability of the compound, whereas a small gap suggests an easy electronic transition [13].

By evaluating the four calculated GAP values (GAP’s 1–4) for each molecule, it is possible to observe low values for Z91 and Z20 compared to the control compounds reinforcing our results in the molecular docking study (see Table 4 and Table 5). Therefore, these results show that all molecules have significant chemical reactivity, necessary for interaction with receptors.

An important evidence (see Table 5) is related to the negative values (in bold) of variations of GAP values (ΔGAP) between the tested and control molecules. These negative values indicate that the GAP values of the molecules tested are lower than the control molecules, pointing out to the higher reactivity of the molecules tested with the ΔGAP values highlighted. This indicates that the antioxidant ability of Z20 and Z91 can be influenced by the four values of GAPs evaluated (transition states 1–4) in the CP450 receptor, by the GAP1 and GAP2 (transition states 1 and 2) in the MP receptor and by GAP1 and GAP3 (transition states 1 and 2) in the NO receptor.

## 3. Materials and Methods

### 3.1. Calculation of Receptor-Ligand Interaction for Evaluation of Antioxidant Potential

Initially, five enzymes (receptors) that respond for the production of reactive oxygen species (ROS) during the metabolism, cytochrome P450 (CP450), lypoxygenase (LO), myeloperoxidase (MP), NADPH oxidase (NO) and xanthine oxidase (XO) were selected and obtained from the Protein Data Bank (PDB) [42]. ΔG values, Receptor-ligand interaction data (binding affinity—BA) and inhibition constant (Ki) values regarding the inhibition of these receptors were obtained in the Autodock 4.2.6/Vina programs [43], respectively, based on standard protocol established by our research group for each analyzed receptor [13,44,45,46,47].

Molecules with positive binding affinity values were not shown, and only molecules with negative binding affinity values were analyzed. A maximum cutoff value of 5.5Å was adopted for the lengths of receptor-ligand interactions [48]. Table 6 shows the protocols used in the validation and subsequent docking analyses of each receptor. The calculations were performed with default parameters by the genetic algorithm, following the protocol described by Pereira et al. and Padilha et al. [49,50].

A total of seven molecules were docked in the five receptors cited, according to Table 1 and Table 7. Caffeine analogs have shown potential inhibitory effect against epithelial cancer according to studies Rogozin et al. [51] that related to the prevention of Epidermal Growth Factor (EGF) in the malignant transformation of epidermal cells of susceptible JB6 rats (P + ) C141 (JB6 P+). In the study carried out by Costa et al. [14], in silico simulations new caffeine-based molecules with potential epithelial anticancer activity have been proposed from the zinc database by virtual screening (ZINC08706191 and ZINC08992920).

The two caffeine analogs used in this in silico study were ZINC08706191 (Z91) and ZINC08992920 (Z20), because the intracellular productions of ROS, which are associated with many cellular events including activation of enzymes [13] and cancer, may be caused by increased oxidative stress mediated by ROS [3,4,5,6]. The inappropriate scavenging of these ROS or enzymatic inhibition can result in degradation of protein, lipid peroxidation, and DNA oxidation, which have been related to skin inflammatory disorders, chronic diseases such as cancer and atherosclerosis besides contributing with the development of degenerative diseases [52,53,54].

### 3.2. Quantum Chemical Calculations

Calculations to obtain molecular descriptors data were performed in vacuum in the Gaussian 09 program for the molecules under study. The method used was the Hartree-Fock in the 6-31G** basis set (HF/6-31G**). The choice of this method was based on the study of Costa et al. (2018) [64], who classified the HF/6-31G** method as the best method for the molecular modeling of caffeine and analogues. The descriptors obtained from calculations were the following: total surface area, molar volume, total dipole moment; molecular hardness (η), molecular softness (1/η), electronegativity (χ), chemical potential (μ), highest occupied molecular orbital energy (HOMO), a level of energy below the highest occupied molecular orbital (HOMO-1), the lowest non-occupied molecular orbital energy (LUMO), and a level of energy above the lowest unoccupied molecular orbital (LUMO+1). GAP values (energy variation between frontier orbitals) were calculated for the four orbitals cited: GAP1 = LUMO − HOMO; GAP2 = LUMO+1 − HOMO; GAP3 = LUMO − HOMO-1; GAP4 = LUMO+1 − HOMO-1.

These data were analyzed and evaluated in relation to their contribution to the antioxidant potential of the tested molecules. Pearson correlation analyzes between the molecular descriptors and Ki values for the studied molecules and the receptors evaluated were performed by the Statistica 6.2 program [65]. Only descriptors with significant values of Ki correlation were shown.

## 4. Conclusions

The study of the antioxidant ability of this article was started from crystallographic data of biological receptors of the said activity available in the Protein Data Bank. These data were important initially to validate the docking protocols of the studied molecules (Z20, Z91, FLU, MEL and DEX). The molecular docking protocols for the five receptors (CP450, MP, LO, NO and XO) were all validated by having RMSD (experimental x theoretical) values lower than 2.0 Å. This allows the certainty that the theoretical data have good agreement with the experimental data.

The binding affinity (BA) was evaluated for poses with lower ΔG values. The LO and XO receptors presented positive values of BA and were excluded from the subsequent analyzes. The excellent correspondence of the negative BA values and the lower Ki values (for the CP450, MP and NO receptors) and the interactions of the molecules Z20 and Z91 in relation to the control molecules and findings of the literature, show good antioxidant ability to the tested molecules.

The highest values of TSA, MV, χ and 1/η for Z91 and Z20, show that larger surface areas and volumes result in a greater number of interactions. Higher electronegativities and 1/η allow these molecules greater capacity to attract electrons from the interactions with the amino acid residues and greater flexibility to twist and adapt to the receptor active site, with a consequent decrease in the Ki values. The lowest values of η and μ, suggest that Z91 and Z20 have a good charge distribution resulting in medium polarity, low stiffness and relationship with thermodynamic properties relative to the quantities of atoms involved in the interactions, and as a consequence also the Ki decrease.

The values of the energies obtained for the molecular orbitals HOMO-1, HOMO, LUMO and LUMO+1 and GAP values, allow the assignment of a strong nucleophilic character (resulting in low Ki values) and high reactivity) to the analyzed molecules (mainly, Z91 and Z20).

The results obtained in this study reveal that Z91 and Z20 have potential antioxidant ability in at least three receptors (CP450, MP and NO) via ROS generation. Thus, Z91 and Z20 may be used for more analyses in order to further evaluate their efficiency in the reduction of oxidative stress and as possible antioxidant to be used in the pharmaceutical industry.

## Figures and Tables

**Table 1:** Structure of the tested molecules used for in silico evaluation of the antioxidant potential.

Molecule	Assignment
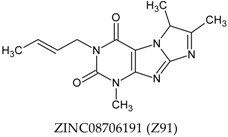	Tested molecule
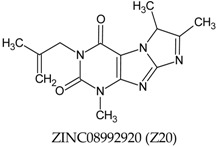	Tested molecule

**Table 2:** Molecular descriptors of the tested (Z20 and Z91) and reference molecules (5-fluorouracil—FLU, melatonin—MEL and dextromethorphan) and their correlations (CKi-CP450, CKi-MP and CKi-NO) with Ki values resulting from the molecular docking study.

**Descriptors**	**Z91**	**Z20**	**FLU**	**MEL**	**DEX**
AST (Å^2^)	457.6300	432.0800	229.5500	438.0500	371.1200
MV (Å^3^)	831.4000	817.9600	348.7100	740.5100	821.6600
χ (eV)	2.9553	2.9776	3.5946	1.9003	1.9594
η (eV)	5.2982	5.3058	6.3539	5.4811	5.9292
1/η (eV)	0.1887	0.1885	0.1574	0.1824	0.1687
μ (eV)	−2.9553	−2.9776	−3.5946	−1.9003	−1.9594
Ki-CP450 (µM)	24.30	17.89	182.0	-	-
Ki-MP (µM)	22.58	19.97	-	185.0	-
Ki-NO (µM)	9.83	3.36	-	-	7.11
**Descriptors**	**CKi-CP450**	**CKi-MP**	**CKi-NO**	**-**	**-**
AST	−0.95	−0.28	−0.51	-	-
VM	−0.99	−0.05	−0.74	-	-
χ	−0.67	−0.60	−0.08	-	-
η	0.85	0.19	0.49	-	-
1/η	−0.83	−0.17	−0.48	-	-
μ	−0.67	−0.60	−0.08	-	-

Å = Angstrom; eV = eletron volt. Significant data at *p* < 0.05.

**Table 3:** Representations of the molecular orbitals and their GAP values (energy variation between frontier orbitals—HOMO and LUMO) between all energy states (^[a]^ GAP_1_, ^[b]^ GAP2, ^[c]^ GAP3 and ^[d]^ GAP4) of the Z20, Z91, 5-fluorouracil (FLU), melatonin (MEL) and dextromethorphan (DEX).

**#**	**LUMO+1**	**LUMO**	**HOMO**	**HOMO** **-** **1**
**Z20**	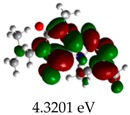	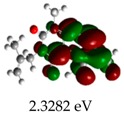	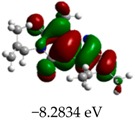	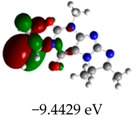
	GAP1 = 10.6116 eV	GAP2 = 12.6035 eV	GAP3 = 11.7711 eV	GAP4 = 13.7630 eV
**Z91**	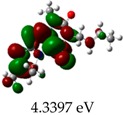	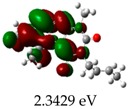	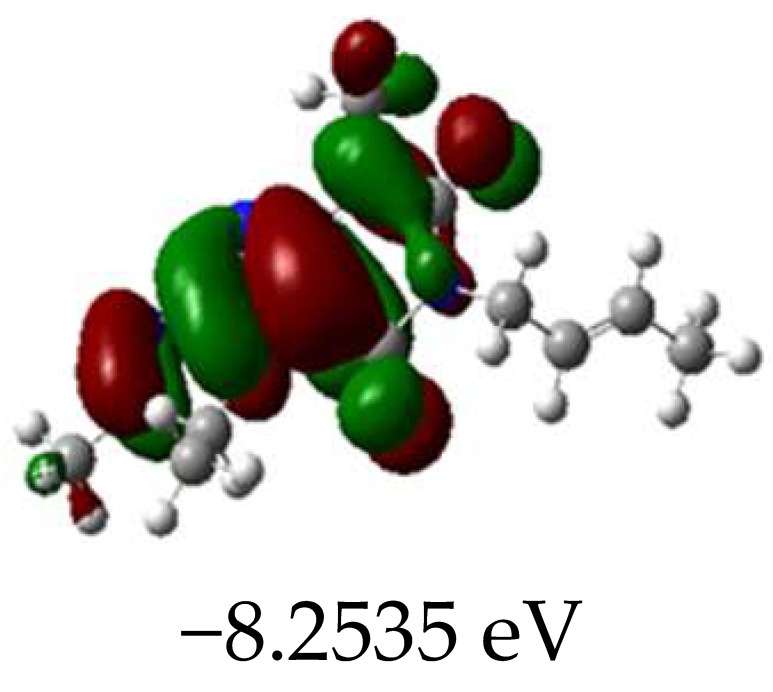	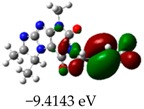
	GAP1 = 10.5964 eV	GAP2 = 12.5932 eV	GAP3 = 11.7572 eV	GAP4 = 13.7540 eV
**FLU**	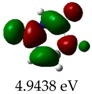	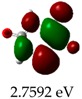	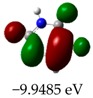	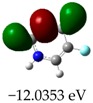
	GAP1 = 12.7077 eV	GAP2 = 14.8923 eV	GAP3 = 14.7945 eV	GAP4 = 16.9791 eV
**MEL**	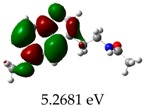	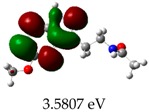	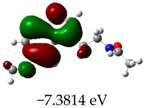	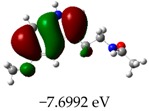
	GAP1 = 10.9621 eV	GAP2 = 12.6495 eV	GAP3 = 11.2799 eV	GAP4 = 12.9853 eV
**DEX**	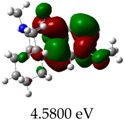	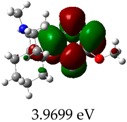	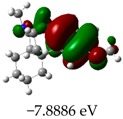	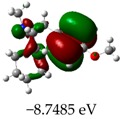
	GAP1 = 11.8585 eV	GAP2 = 12.4686 eV	GAP3 = 12.4479 eV	GAP4 = 13.3285 eV

^[a]^ GAP1 = LUMO − HOMO; ^[b]^ GAP2 = LUMO+1 − HOMO; ^[c]^ GAP3 =LUMO − HOMO-1; ^[d]^ GAP4 = LUMO+1 − HOMO-1.

**Table 4:** Correlations between molecular orbitals data and Ki values.

Descriptors	Enzymes		
	CP450	MP	NO
HOMO-1 (eV)	−0.38	−0.81	−0.92
HOMO (eV)	−0.13	−0.47	−0.22
LUMO (eV)	0.91	0.54	0.30
LUMO+1 (eV)	0.88	0.60	0.23

**Table 5:** Variations of GAP values compared to controls.

ΔGAP	GAP1 *	GAP2 *	GAP3 *	GAP4 *
ΔGAP (Z20-FLU)	−2.0961	−2.2888	−3.0234	−3.2161
ΔGAP (Z91-FLU)	−2.1113	−2.2991	−3.0373	−3.2251
ΔGAP (Z20-MEL)	−0.3505	−0.3586	0.4912	0.7777
ΔGAP (Z91-MEL)	−0.3657	−0.3689	0.4773	0.7687
ΔGAP (Z20-DEX)	−1.2469	0.1349	−0.6768	0.4345
ΔGAP (Z91-DEX)	−1.2621	0.1246	−0.6907	0.4255

* Tested molecule GAP—Control GAP.

**Table 6:** Data from protocols used in the molecular docking validation.

Receptor	Ligand	Coordinates of the Grid Center	Grid Size (points)
CP450 (PDB code: 1OG5)	*S*-Warfarin	−20.257x 86.991y 38.581z	22 x 20 y 24 z
LO (PDB code: 1N8Q)	Protocatecuic acid	21.864x 2.184y 18.909z	24 x 18 y 12 z
MP (PDB code: 1DNU)	N-Acetyl-D-glucosamine	39.817x −38.635y −5.308z	22 x 30 y 18 z
NO (PDB code: 2CDU)	Adenosine-5’-diphosphate	1.687x 9.885y 54.962z	30 x 14 y 32 z
XO (PDB code: 3NRZ)	Hypoxanthine	89.018x 9.4501y 18.290z	37 x 37 y 37 z

**Table 7:** Structure of the controls molecules used for in silico evaluation of the antioxidant potential.

Molecule	Assignment
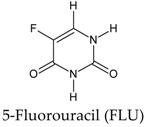	Molecule (control) present in commercially available drug with inhibitory activity at the CP450 receptor [55,56].
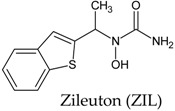	Molecule (control) present in commercially available drug with inhibitory activity at the LO receptor [56,57,58,59].
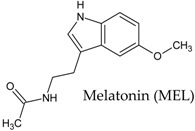	Molecule (control) present in commercially available drug with inhibitory activity at the MP receptor [56,59,60].
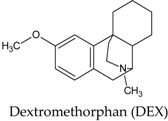	Molecule (control) present in commercially available drug with inhibitory activity at the NO receptor [56,61,62].
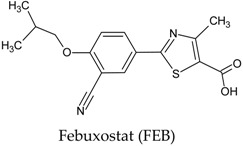	Molecule (control) present in commercially available drug with inhibitory activity at the XO receptor [13,56,63].
